# Lessons Learned About the Implementation and Sustainability of a Community‐Driven Asthma Intervention in Public Schools

**DOI:** 10.1111/josh.70229

**Published:** 2026-09-08

**Authors:** Liza Tomczuk, Melanie Pellecchia, Freya Nezir, Hanna Christian, Tyra Bryant‐Stephens, Courtney Benjamin Wolk

**Affiliations:** ^1^ Dornsife School of Public Health Drexel University Philadelphia Pennsylvania USA; ^2^ Perelman School of Medicine, University of Pennsylvania Philadelphia Pennsylvania USA; ^3^ Children's Hospital of Philadelphia Philadelphia Pennsylvania USA

**Keywords:** access to care, asthma, community health workers, implementation, sustainability

## Abstract

**Background:**

Racially minoritized and economically disadvantaged children are disproportionately affected by asthma and often receive less or lower quality care. This paper describes the implementation of a community health worker (CHW)‐delivered program in the School District of Philadelphia, asthma management challenges in under‐resourced schools, and provides potential solutions.

**Contributions to Practice:**

Implementation outcomes for two school‐based interventions were evaluated to inform sustainment. Ten school nurses and administrators participated in semi‐structured interviews about intervention experience, sustainment, resource gaps, and supports needed to continue the interventions. The research team observed intervention delivery and rated fidelity. Medication management, education, and coordination costs for nurses and CHWs were estimated and annualized over a 180‐day school year. Interviews identified five themes: (1) barriers, (2) facilitators, (3) recommendations, (4) successes/impacts, and (5) sustainment. Lesson fidelity was measured across 7 schools and varied across lessons. CHW (≈$3366/school) and nurse costs (≈$1620/school) differed by over $70,000.

**Implications for School Health Policy, Practice, and Equity:**

Care coordination, community partnerships, and reimbursement for CHW‐supported programs in public schools should be instituted to implement and sustain asthma interventions and improve care access for racially minoritized and economically disadvantaged children.

**Conclusions:**

Despite nurses' and/or administrators' satisfaction with the intervention, it was not sustainable without CHWs.

Asthma is highly prevalent [1] and disproportionately affects racially minoritized and economically disadvantaged school‐aged children in urban areas [2, 3, 4, 5]. Specifically, asthma occurs more often in Black and Hispanic children compared to white children, as well as children residing in communities with fewer socioeconomic resources compared to those living in communities with less poverty [2]. These youth are often undertreated and encounter barriers to accessing evidence‐based care [6]. In addition to detrimental health effects, recent estimates suggest that the financial cost of asthma in the United States exceeds $81 billion every year [3]. Cost‐effective solutions to increase access to effective asthma care for racially minoritized and economically disadvantaged youth must be deployed to improve health outcomes.

Public schools represent an important context for supporting youth with asthma because they can serve as accessible locations for youth to receive health education and supports. Evidence‐based programs exist to support asthma care in schools, including those leveraging community health workers (CHWs) [7]. Understanding factors associated with the implementation and sustainment of school‐based asthma programs in public schools is necessary for developing appropriate and accessible youth asthma care options that can be maintained over time.

This evaluation aimed to elucidate what did and did not work in the implementation of two CHW‐delivered asthma interventions in schools, key challenges associated with asthma management in under‐resourced schools, and suggestions for future adopters of similar programs.

## 1 Presentation of the Practice

The West Philadelphia Asthma Control Collaborative (WEPACC) study evaluated two evidence‐based interventions to integrate home, school, healthcare, and community asthma supports for children ages 5–13 in an under‐resourced urban area: [8] (1) Yes We Can, a primary care intervention with a home visitation component and (2) Open Airways for Schools Plus (OAS Plus) and School Based Asthma Therapy (SBAT) [8]. In this perspective piece, we evaluated implementation outcomes to inform sustainment of the school‐based interventions (OAS Plus and SBAT) because their implementation with CHWs in schools was novel. OAS Plus included six asthma management educational lessons and guidance to nurses on medication administration. Lessons focused on identifying asthma triggers, recognizing warning signs, following an Asthma Action Plan, and stress management, delivered by CHWs. The SBAT intervention delivered asthma medication to students. Further details of the interventions and their implementation are described in the larger study [9].

Evaluation activities associated with OAS Plus and SBAT intervention assessment were oriented toward understanding implementation and sustainment processes and outcomes to inform future practice and policy. All administrators and the CHWs and nurses administering OAS Plus and SBAT from participating schools were eligible to participate in interviews. Study activities were Institutional Review Board approved, and interview participants provided informed consent.

### 1.1 Participating Schools and Personnel

Public elementary and charter schools in West Philadelphia were invited to participate in the larger study. Philadelphia is the poorest of the nation's 10 largest cities, with approximately 20.3% of its residents living in poverty [10]. The poverty rate in West Philadelphia is even higher, with estimates suggesting that over 30% of residents in this section of the city live in poverty [11]. A total of 33 nurses and school administrators from 16 West Philadelphia schools participating in the larger study were then invited to participate in semi‐structured interviews following implementation of the school‐based interventions. Ten school nurses and administrators (i.e., principals, assistant principals, education coordinators) from the larger study participated in one‐time virtual, semi‐structured individual interviews from February to April 2023. Nurse and administrator interview participants came from 9 different public schools in Philadelphia. Intervention fidelity was assessed among five CHWs from 7 unique schools from the larger study.

### 1.2 Evaluation Tools and Procedures

#### 1.2.1 Qualitative Interviews

A semi‐structured interview guide was drafted, guided by the Exploration, Preparation, Implementation, and Sustainment (EPIS) framework [12]. Semi‐structured interviews were completed with 10 school nurses and administrators with experience implementing OAS Plus and SBAT. Nurses and administrators were queried about their experience implementing the CHW‐supported interventions for students with asthma, how, if at all, schools were sustaining program components following study end, financial issues or resource gaps experienced, and supports needed to continue OAS Plus and SBAT delivery. Interviews lasted approximately 16–51 min and occurred after OAS Plus and SBAT implementation concluded.

Interview data was analyzed using a rapid qualitative analysis procedure. Interviewers took notes during virtual interviews and completed a structured summary sheet immediately following the interview to document important information about the participant's role in intervention delivery and sustainment, OAS Plus and SBAT implementation and sustainment barriers, facilitators, and challenges, communication surrounding intervention sustainment, and stakeholder recommendations. This information was transferred to an Excel data matrix for synthesis and identification of key themes, learnings, and recommendations.

#### 1.2.2 Intervention Fidelity

Intervention fidelity was assessed using an established fidelity tool specific to each of the six lessons [9]. Fidelity items focused on discrete aspects of the lesson, such as covering session objectives in sufficient detail, engaging students in planned learning activities, summarizing session content, and items related to student attendance and attention and classroom supplies and facilities. Five CHWs were observed a total of 52 times across 7 schools. Aggregate mean intervener fidelity was calculated per lesson (Lesson 1 = 82%; Lesson 2 = 79%; Lesson 3 = 66%; Lesson 4 = 61%; Lesson 5 = 62%; Lesson 6 = 57%). Descriptive and summary statistics were calculated for intervention fidelity for observed lessons. Fidelity was calculated as a percentage of overall components scored as observed (i.e., scored “yes”) during each observation. Mean overall fidelity for each lesson was calculated across observations for each lesson.

#### 1.2.3 Intervention Cost

Time allocation estimates for core intervention activities—such as medication management, educational delivery, and related coordination—were obtained by personnel role for a school year (180 instructional days) through interviews with key staff. Reported time use for each activity, by personnel category, was converted into annual hours per school based on an 180‐day school year. These hours were multiplied by the national average hourly wage for each personnel category, as reported by the US Bureau of Labor Statistics, with an additional 30% fringe benefit rate applied. Activity‐level costs were then aggregated to produce annual per‐school totals by personnel category, which are reported descriptively.

## 2 Evaluation Results

### 2.1 Qualitative Interview Findings

We interviewed six school nurses, two administrators, an assistant principal, and a program coordinator. See Table 1 for interviewee demographic characteristics. Five overarching themes emerged from interviews about the implementation and sustainment of OAS Plus and SBAT: (1) barriers, (2) facilitators, (3) recommendations, (4) successes/impacts, and (5) sustainment.

**TABLE 1:** Sustainment phase interviews demographic characteristics (*N* = 10).

Variable	No. (%)
Mean age (SD)	49.8 (16.9); Range: 28–73
Sex	
Male	1 (10)
Female	7 (70)
Non‐binary	1 (10)
Prefer not to disclose	1 (10)
Ethnicity	
Hispanic/Latino	0 (0)
Non‐Hispanic and/or non‐Latino	10 (100)
Missing	0 (0)
Race	
American Indian/Alaska native	1 (10)
Asian	0 (0)
Black or African American	4 (40)
Native Hawaiian or other Pacific islander	0 (0)
White	5 (50)
Multiple races	0 (0)
Missing	0 (0)
Role	
Nurse	6 (60)
Administrator	2 (20)
Other	2 (20)
Program coordinator	1 (10)
Assistant principal	1 (10)

*Barriers* to implementation included nurses' workloads, difficulty scheduling students, school environmental triggers, and the interventions being viewed as low priority compared to other competing demands. One school nurse said, “*There is so much else on our plates as nurses, especially because the climate is becoming more dangerous with fights so a lot of my time is spent with that, and we still have state‐mandated screenings so have to do those*…” Beliefs about the caregiver's role in medication administration, parental knowledge about asthma, difficulty getting inhalers and medication into school, and challenges getting required medical forms completed by caregivers were also described as barriers.

*Facilitators* included reduction in environmental triggers within schools (i.e., new ventilation system) and staff desire to receive asthma education. A school nurse that was present during intervention implementation stated, “…*I feel the students received the benefit and I did too from having the ambassador here for SBAT and the classes, and we had a training for the staff – all of these things were beneficial and I would hate to see them totally leave us – if we can somehow get these things back in, even if tweaked and not on the same scale, just start to get some pieces back in place to get some instruction and support to students and families*.”

CHW characteristics (i.e., CHW's positive regard toward students and parents, initiative, communication) were also described as facilitators to OAS Plus and SBAT implementation. A key facilitator was that the CHWs were not employed directly by the schools. Instead, they were employed by the research team and supervised by school nurses. One school administrator spoke to the benefits of this approach: “*It was nice when someone would come in because they had a specific time when kids would come down and do their medicine. This was helpful for the school nurse because she has a ton of other things to worry about*.” This model ensured costs were not incurred by the schools, reducing financial barriers.

*Recommendations* related to improving administrative processes and intervention delivery. One interviewee recommended medical form completion occur during pediatric well visits. School recommendations included incorporating intervention delivery into existing health education in schools, expanding reach to more students, and increasing staffing.

#### 2.1.1 Success and Impacts

School staff perceived the intervention as beneficial to students and that it provided tools to minimize environmental triggers. Asthma education was perceived as important by both staff and CHWs. “*OAS was really valuable to teaching students and their siblings too, our teachers really appreciated the PD* [Professional Development]. *It wasn't long, it was short and to‐the‐point and helpful. Teachers loved getting supplies to take care of their classrooms for kids with asthma*,” one interviewee noted.

#### 2.1.2 Sustainment

Participants discussed that the interventions were not sustained, primarily due to the absence of CHW once the study was completed. Nurses and administrators expressed satisfaction and eagerness to sustain the interventions but needed more resources and guidance. Staff turnover and vacant nursing positions were also reported as impacting sustainability.

### 2.2 Intervention Fidelity

Five CHWs were observed 52 times across 7 schools. Fidelity (% of core components observed) varied across OAS lessons: Lesson 1 = 82%; Lesson 2 = 79%; Lesson 3 = 66%; Lesson 4 = 61%; Lesson 5 = 62%; Lesson 6 = 57%. Fidelity ranged from 15% to 93% across lessons.

### 2.3 Intervention Cost

Over 180 school days, nurses spent 27 hours (h) on the intervention annually/school, including 3 h/year for scheduling and coordination. With 27 h of nurse time at a national average wage of $46/h plus 30% fringe benefits—totaling $60/h—the nurse's time cost associated with these interventions was approximately $1620/school.

CHWs dedicated 30 h annually to coordination and communication tasks, including scheduling classes, coordinating with parents, ensuring medication availability at school, and eligibility screenings. When factoring in an additional 48 h annually for delivering educational lessons, CHW's total time commitment was 102 h per year. At a national average wage of $25/h plus 30% fringe benefits—totaling $33/h—the CHW's time cost was ≈$3366/school. During the study, schools did not bear the cost of CHWs, reducing the financial burden on schools. In the sustainment phase, CHWs only did initial coordination and could manage up to 10 schools/year.

## 3 Contributions to Practice

This evaluation of two school‐based, CHW‐delivered interventions yielded findings imperative for advancing and sustaining asthma care in urban public schools. Semi‐structured interviews with school nurses and administrators identified key barriers and facilitators to success, recommendations for program improvement, successes and impacts of the interventions, and sustainment challenges. Intervention fidelity varied across lessons, highlighting variability in how the content was implemented in schools. CHW and nurse costs were modest, but these costs may nonetheless be challenging for low‐resource schools to absorb in the absence of external support. Taken together, these findings provide insight into potential improvements to intervention implementation and sustainment over time that can improve access to asthma care for children attending under‐resourced schools.

### 3.1 Implications for School Health Research

Public schools are novel settings for implementation of CHW‐delivered asthma interventions for school‐aged children in urban areas. Increased workloads for school nurses and limited time and resources were barriers to sustainment, though interviewees also recognized the importance of the intervention. Although interviewees expressed satisfaction, the intervention was not sustainable without CHWs. Future school health research should continue to explore alternatives to school nurse‐delivered asthma interventions.

Implementation and sustainment of school‐based asthma interventions require strategic use of resources. Embedding asthma education into existing health curricula could reduce costs and ensure sustainability. Funding, such as insurance reimbursements for CHW services or partnerships with public health agencies, could also offset expenses. School health research should prioritize streamlining CHW activities by training school staff in environmental assessments or integrating asthma education into standard curricula, which could further reduce costs and support sustainability.

### 3.2 Implications for School Health Policy, Practice, and Equity

The findings from this study have direct implications for school health policy, practice, and equity. CHWs were essential to the implementation of these school‐based programs and represent a relatively low‐cost staffing model that can be used to support the delivery of evidence‐based practices for asthma in schools and long‐term sustainment of these practices. Policies that support the adoption of CHWs in schools can improve health outcomes for children with asthma from minoritized groups and reduce healthcare disparities. Therefore, school district leaders and health professionals should consider utilizing this asthma treatment model in schools across the country.

## 4 Limitations

There are several limitations of the study described in this paper that must be acknowledged. Due to the pandemic, only 114 out of an anticipated 600 children received the school intervention as intended due to school closures and the inability of the intervention to be delivered to students. Therefore, the fidelity assessment was conducted at the beginning of the study and only in some of the schools. This project was conducted in a single urban area (West Philadelphia), so the results may not generalize to other school districts or regions.

## 5 Conclusions

Racially minoritized and economically disadvantaged children are at increased risk of severe asthma, and efforts to increase access to asthma care must be taken. The inability to sustain a community‐driven asthma intervention in under‐resourced schools highlights the critical need to better support school‐administered asthma interventions. Care coordination must be enhanced to increase access to treatment and adherence to medications [13]. Additionally, community partnerships should be utilized to fill gaps in existing service systems [14]. Reimbursement for CHW‐supported programs in schools should be instituted to implement and sustain asthma interventions.

## Funding

This work was supported by the National Heart, Lung, and Blood Institute, U01HL138687.

## Ethics Statement

The work discussed in this manuscript required and received IRB approval from the University of Pennsylvania and the Children's Hospital of Philadelphia Institutional Review Boards.

## Conflicts of Interest

The authors declare no conflicts of interest.

## Data Availability

The data that support the findings of this study are available from the corresponding author upon reasonable request.
